# Stimulation of Sphingosine Kinase 1 (SPHK1) Is Beneficial in a Huntington’s Disease Pre-clinical Model

**DOI:** 10.3389/fnmol.2019.00100

**Published:** 2019-04-24

**Authors:** Alba Di Pardo, Giuseppe Pepe, Salvatore Castaldo, Federico Marracino, Luca Capocci, Enrico Amico, Michele Madonna, Susy Giova, Se Kyoo Jeong, Bu-Mahn Park, Byeong Deog Park, Vittorio Maglione

**Affiliations:** ^1^IRCCS, Neuromed, Pozzilli, Italy; ^2^Department of Cosmetic Science, Seowon University, Cheongju, South Korea; ^3^NeoPharm USA Inc., Engelwood Cliffs, NJ, United States; ^4^Dr. Raymond Laboratories, Inc., Englewood Cliffs, NJ, United States

**Keywords:** HD, K6PC-5, SPHK1, aggregates, neuroprotection

## Abstract

Although several agents have been identified to provide therapeutic benefits in Huntington disease (HD), the number of conventionally used treatments remains limited and only symptomatic. Thus, it is plausible that the need to identify new therapeutic targets for the development of alternative and more effective treatments is becoming increasingly urgent. Recently, the sphingosine-1-phosphate (S1P) axis has been reported to be a valid potential novel molecular target for therapy development in HD. Modulation of aberrant metabolism of S1P in HD has been proved to exert neuroprotective action *in vitro* settings including human HD iPSC-derived neurons. In this study, we investigated whether promoting S1P production by stimulating Sphingosine Kinase 1 (SPHK1) by the selective activator, K6PC-5, may have therapeutic benefit *in vivo* in R6/2 HD mouse model. Our findings indicate that chronic administration of 0.05 mg/kg K6PC-5 exerted an overall beneficial effect in R6/2 mice. It significantly slowed down the progressive motor deficit associated with disease progression, modulated S1P metabolism, evoked the activation of pro-survival pathways and markedly reduced the toxic mutant huntingtin (mHtt) aggregation. These results suggest that K6PC-5 may represent a future therapeutic option in HD and may potentially counteract the perturbed brain function induced by deregulated S1P pathways.

## Introduction

Huntington’s disease (HD) is a fatal inherited brain disorder characterized by progressive striatal and cortical neurodegeneration and associated motor, cognitive and behavioral disturbances (McColgan and Tabrizi, 2017). The disease results from the expansion of a polyglutamine stretch (polyQ; >36 repeats) in the N-terminal region of huntingtin (Htt), a widely expressed protein whose function is still under investigation (Jimenez-Sanchez et al., 2017).

Expansion of the polyQ tract endows mutant Htt (mHtt) with toxic properties, resulting in the development of a number of deleterious effects in both neuronal and non-neuronal cells (Maglione et al., 2005, 2006a,b; Carroll et al., 2015; Jimenez-Sanchez et al., 2017). Defects in the metabolism of sphingosine-1-phosphate (S1P) have recently emerged as an important factor in the disease pathogenesis (Di Pardo et al., 2017a,b; Pirhaji et al., 2017).

S1P is one the most potent signaling lipids that regulates several molecular events underlying cellular homeostasis and viability (Maceyka et al., 2012) and whose homeostasis is finely governed by the action of number of different highly specialized enzymes (Le Stunff et al., 2002; Morozov et al., 2013). Reduction of S1P levels is associated with different neurodegenerative disorders (Di Pardo and Maglione, 2018b) only recently including HD (Pirhaji et al., 2016, 2017; Di Pardo et al., 2017a,b). Although the molecular mechanism behind the reduction of S1P content is thought to be complex in the case of HD it may be in part due to the reduced levels of the S1P biosynthetic enzyme, sphingosine kinase-1 (SPHK1; Di Pardo et al., 2017a,b), whose activity is normally associated with cell survival (Le Stunff et al., 2002; Morozov et al., 2013). In line with that, stimulation of SPHK1, with the selective activator K6PC-5, exerts beneficial effects and pro-survival actions in *in vitro* models of HD, and importantly also in human iPSC-derived neurons from HD patients (Di Pardo et al., 2017a).

In this study, we demonstrate for the first time that stimulation of SPHK1 is therapeutically effective in R6/2 mice.

The R6/2 mouse model, overexpressing the exon 1 of the human HD gene (*HTT*) with long (141–157) CAG-repeat expansions (Mangiarini et al., 1996), is one of the best-characterized and the most widely used animal models to study the pathogenesis of HD. Motor symptoms usually start at 6 weeks of age and progressively worsen over weeks (Mangiarini et al., 1996; Di Pardo et al., 2014). Despite the wealth of different transgenic HD mice available, the R6/2 line remains one of the most used models for testing novel therapeutic interventions for HD, and many compounds that have been reported effective in these mice have proceeded to clinical trials with mixed results (Chang et al., 2015).

Our data indicate that chronic administration of K6PC-5 exerts an overall therapeutic action in R6/2 mice with amelioration of the forelimb and hindlimb clasping, and prevention of motor deficit progress. Interestingly, from a mechanistic perspective, the treatment with K6PC-5 stimulates S1P metabolism, evokes the activation of pro-survival pathways and autophagic flux and reduces mHtt toxicity which translates into a significant slow-down of the overall disease progression in HD mice.

## Materials and Methods

### Animals

Breeding pairs of the R6/2 line of transgenic mice [strain name: B6CBA-tgN (HDexon1) 62Gpb/1J] with 160 (CAG) repeat expansions were purchased from the Jackson Laboratories and were crossed with female B6CBA wild-type (WT) mice to establish the animal colony. All experimental procedures were approved by the IRCCS Neuromed Animal Care Review Board and by “Istituto Superiore di Sanità” (ISS permit number: 1163/2015-PR) and were conducted according to the 2010/63/EU Directive for animal experiments.

Analyses were carried out in both R6/2 mice and WT littermates, starting from 5 weeks of age. To ensure homogeneity of the experimental cohorts, mice from the same F generation were assigned to experimental groups, such that age and weight were matched.

### *In vivo* Drug Administration

K6PC-5 (provided by NeoPharm) was dissolved in DMSO, further diluted in saline (vehicle) and daily administered, starting at 6 weeks of age, by intraperitoneal (i.p.) injection at a dose of 0.05 mg/kg, whose effectiveness was determined by an explorative pilot study (Supplementary Figure S1). Control mice (WT and R6/2) were injected daily with the same volume of vehicle containing DMSO.

### Clasping Analysis

The clasping score is determined over 30 s. In particular, mice were suspended by their tails from a height of 50 cm and a limb-clasping response was defined as the withdrawal of any limb to the torso for more than 2 s. The following scores were used: 0, absence of clasping; 0.5, withdrawal of any single limb; 1, withdrawal of any two limbs; 1.5, withdrawal of any three limbs; 2, withdrawal of all four limbs.

### Motor Behavior Tests

Motor performance was assessed by the Horizontal Ladder Task and Rotarod tests as described previously (Di Pardo et al., 2018). All tests took place during the light phase of the light-dark cycle and mice were tested before and after the initiation of the treatment as indicated.

All animals used for biochemical and histological experiments were euthanized after 5 weeks of treatment (11 weeks of age). Mice used for life-span analysis were examined once daily until natural death.

### Brain Lysate Preparation

Eleven-week-old mice were sacrificed within 1 h from the last treatment by cervical dislocation and brains were removed from the skull, weighed and dissected. Brains were immediately snap-frozen in liquid N2 and pulverized in a mortar with a pestle as previously described (Di Pardo et al., 2018).

### Semi-quantitative Analysis of Sphingosine-1-Phosphate (S1P)

To assure that an equal amount of homogenate was analyzed, each tissue lysate sample was serially diluted, and the protein concentration was assessed by NanoDrop Spectrophotometer. Five-hundred nanograms of striatal lysate from vehicle- and K6PC-5 treated R6/2 mice were spotted in quadruplicate on a nitrocellulose membrane. Membranes were incubated with the anti-S1P antibody (LT1002; 1:500; Echelon Biosciences, Cat. N. Z-P300), and with the anti-Actin antibody (1: 5,000; Sigma Aldrich, Cat. N. A5441; see Supplementary Material). S1P- and actin-immunopositive spots were visualized by ECL Plus (GE Healthcare) and quantitated with Image Lab Software (Bio-Rad Laboratories).

### Quantitative Real Time PCR (qPCR)

Mice were sacrificed by cervical dislocation and brain regions were dissected out, snap frozen in liquid N2 and pulverized in a mortar with a pestle. Total RNA was extracted using the RNeasy kit (Qiagen) according to the manufacturer’s instructions. One microgram of total RNA was reverse-transcribed using Superscript II reverse transcriptase (Invitrogen) and oligo-dT primer, and the resulting cDNAs were amplified using Sso Advanced Universal SYBR Green Supermix (Bio-Rad Laboratories, Cat. N. #1725271) following the manufacturers’ instructions. Quantitative PCR (qPCR) analysis was performed on a CFX Connect Real-Time System instrument (Bio-Rad Laboratories) as previously described (Di Pardo et al., 2017b). The following primers were used (5′→3′): CDase-Fw: GTGTGGCATATTCTCATCTG; CDase-Rv: TAAGGGACACCAATAAAAGC. CerS1-Fw: CACACATCTTTCGGCCCCT; CerS1-Rv: GCGGGTCATGGAAGAAAGGA; CerS2-Fw: GGTGGAGGTAGACCTTTTGTCA; CerS2-Rv: CGGAACTTTTTGAGAAGACTGGG; sphk1-Fw: ACAGTGGGCACCTTCTTTC; sphk1-Rv: CTTCTGCACCAGTGTAGAGGC. Expression of all sphingolipid-metabolizing enzymes was normalized on Cyclophilin-A by using the following primers: CycA-Fw: TCCAAAGACAGCAGAAAACTTTCG; CycA-Rv: TCTTCTTGCTGGTCTTGCCATTCC.

### Immunoblottings

Proteins (20 μg) were resolved on 10% SDS-PAGE and immunoblotted with the following antibodies: anti-S1PR_1_ (1:1,000; Immunological Sciences, Cat. N. AB-83739); anti-S1PR_5_ (1:1,000; Immunological Sciences, Cat. N. AB-83741); anti-phospho-AKT (1:1,000; Immunological Sciences, Cat. N. AB-10521); anti-AKT (1:1,000; Cell Signaling Cat. N. #2920), anti-phospho-ERK (1:1,000; Immunological Sciences, Cat. N. AB-82379); anti-dopamine- and cAMP-regulated protein 32 (DARPP-32; 1:1,000; Cell Signaling, Cat. N. #2302); anti-brain derived neurotrophic factor (BDNF; 1:1,000; Santa Cruz, Cat. N. sc-546).

For LC3 and Beclin1 analyses, protein lysates (20 μg) were resolved on a 12% SDS-PAGE and immunoblotted with anti-LC3 (1:1,000; Novus, Cat. N. NB100-2331) and anti-Beclin1 (1:1,000; Santa Cruz, Cat. N. sc-11427) antibodies. For protein normalization, anti-Actin (1: 5,000; Sigma Aldrich, Cat. N. A5441) and/or anti-Cyclophilin (Abcam Cat. N. ab16045) were used. Immunoblots were then exposed to specific HRP-conjugated secondary antibodies (Santa Cruz, Cat. N. sc-2004 and sc-2005). Protein bands were visualized by ECL Plus (GE Healthcare) and quantitated with Image Lab Software (Bio-Rad Laboratories). Cell lysate from HUVEC, treated with the autophagy inductor TAT-D11 (10 μM; Shoji-Kawata et al., 2013) was used as positive control.

### Analysis of mHtt Aggregates

WT and R6/2 mice were sacrificed by cervical dislocation. Brains were removed and trimmed by removing the olfactory bulbs and spinal cord. The remaining brain was processed and embedded in paraffin wax and 10 μg coronal sections were cut. Four mice/group were used and immunostaining for mHtt aggregates was carried out using a mouse anti-Htt antibody (clone EM48; 1: 150; Immunological Sciences, Cat. N. MAB-94354) as recently described (Di Pardo et al., 2018). For the immunoblotting analyses, cell lysate (30 μg) was resolved on a 10% SDS-PAGE, and entire gel, including the stacking portion, was transblotted over-night at 250 mV in 0.05% SDS and 16% methanol-containing transfer buffer (Di Pardo et al., 2018). The membrane was blocked in 5% non-fat dry milk TBST for 1 h and successively immunoblotted with anti-Htt (clone EM48) antibody (1:1,000). A monoclonal anti-mouse HRP-conjugated antibody (Santa Cruz, Cat. N. sc-2005) was used as a secondary antibody. Protein bands were visualized by ECL Plus (GE Healthcare) and quantified as described above.

### Statistics

A Two-way ANOVA followed by a Bonferroni post-test was used to compare treatment groups for the Horizontal Ladder Task and Rotarod tests, as well as for the mouse body weight analysis. A Log-rank test was used to analyze mouse survival. A One-way ANOVA and Two-tailed Unpaired *t*-test was used in all other experiments as indicated. All data were expressed as mean ± SD.

## Results

### Treatment With K6PC-5 Prevents Motor Dysfunction in R6/2 Mice

K6PC-5 treatment has previously been demonstrated to activate pro-survival pathways in *in vitro* models of HD including human HD patient iPSC-derived neurons (Di Pardo et al., 2017a), however, whether the compound could exert any therapeutic action *in vivo* has never been investigated so far. Here, in order to test the therapeutic potential of K6PC-5, whose potential ability to cross the blood-brain barrier was postulated *in vitro* by the PAMPA assay (see Supplementary Figure S2), symptomatic R6/2 mice (6-week-old) and age-matched WT littermate controls were i.p.- injected with 0.05 mg/kg daily, and limb clasping reflex and motor phenotype with movement and coordination were then analyzed.

**Figure 1:**
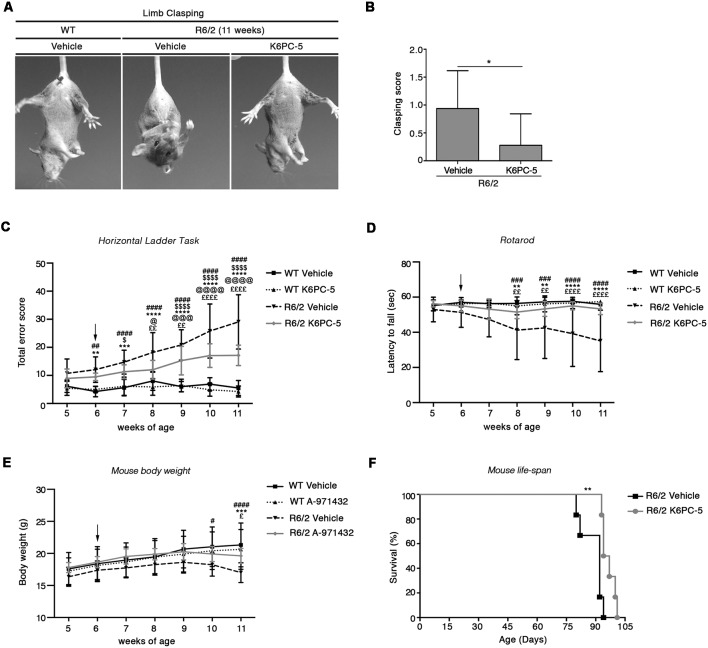
Administration of K6PC-5 ameliorates Huntington’s disease (HD) mouse phenotype. **(A,B)** Limb-clasping response at 11 weeks of age HD. Vehicle-treated R6/2 mice, *N* = 8; K6PC-5-treated R6/2 mice, *N* = 9. Values are represented as mean ± SD. *p < 0.05 (Un-paired *t*-test). Motor performance assessed by **(C)** Horizontal Ladder Task and **(D)** Rotarod. Vehicle-treated wild-type (WT) mice, *N* = 8; K6PC-5-treated WT mice, *N* = 8; vehicle-treated R6/2 mice, *N* = 15; K6PC-5-treated R6/2 mice, *N* = 15. **(E)** Mouse body weight during the entire period of the treatment. Vehicle-treated WT mice, *N* = 8; K6PC-5-treated WT mice, *N* = 8; vehicle-treated R6/2 mice, *N* = 12; K6PC-5-treated R6/2 mice, *N* = 12. Values are represented as mean ± SD. ^#^*p* < 0.05; ^##^*p* < 0.01; ^###^*p* < 0.0001; ^####^*p* < 0.0001 (vehicle-treated WT vs. vehicle-treated R6/2 mice). ^$^*p* < 0.05; ^$$$$^*p* < 0.0001 (vehicle-treated WT vs. K6PC-5-treated R6/2 mice). **p < 0.01; ***p < 0.001; ****P < 0.0001 (K6PC-5-treated WT vs. vehicle-treated R6/2 mice). ^@^*p* < 0.05; ^@@@^*p* < 0.001; ^@@@@^*p* < 0.0001 (K6PC-5-treated WT vs. K6PC-5-treated R6/2 mice). ^£^*p* < 0.05; ^££^*p* < 0.01; ^££££^*p* < 0.0001 (vehicle-treated R6/2 vs. K6PC-5-treated R6/2 mice; Two-Way ANOVA with Bonferroni post-test). **(F)** Kaplan Maier curve of survival HD. Vehicle-treated R6/2 mice, *N* = 6; K6PC-5-treated R6/2 mice, *N* = 6. **p < 0.01. Log-rank (Mantel-Cox) test.

In contrast to what classically occurs as the disease progresses, R6/2 mice treated with K6PC-5 did not exhibit clasping behavior even at the late stage of the disease (Figures 1A,B). K6PC-5 was also therapeutically effective in preventing the worsening of motor functions in these mice. R6/2 mice treated with the compound performed significantly better than vehicle-treated mice during the whole period of the treatment as assessed by Horizontal Ladder Task and Rotarod (Figures 1C,D). Interestingly, the beneficial effect of K6PC-5 administration was also detectable when animal body weight (Figure 1E) and animal life-span (Figure 1F) were assessed. No evidence of adverse effects was observed throughout the period of the treatment.

### K6PC-5 Treatment Modulates S1P Metabolism/Axis in HD Mice

Over the last few years, we have extensively demonstrated that the metabolism of different sphingolipids, including S1P, is aberrant in different HD settings ranging from pre-clinical models to human samples from HD patients (Maglione et al., 2010; Di Pardo et al., 2016, 2017a,b; Di Pardo and Maglione, 2017).

**Figure 2:**
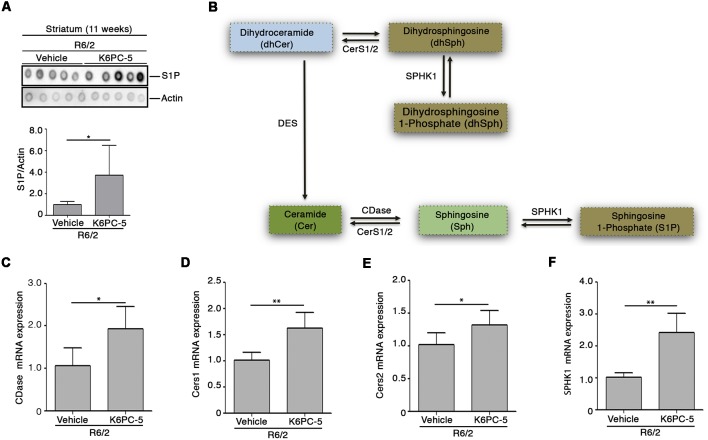
K6PC-5 modulates sphingosine-1-phosphate (S1P) metabolism in the striatum of R6/2 mice.** (A)** Representative cropped dot blotting and densitometric analysis of S1P content in striatal tissues from vehicle- and K6PC-5-treated R6/2 mice at 11 weeks of age. Each spot represents a single animal. Graph represents the average of *N* = 7 vehicle-treated and *N* = 5 K6PC-5-treated R6/2 mice. Data are represented as mean ± SD. *p < 0.05 (Unpaired *t*-test). **(B)** Simplified schematic representation of sphingolipid biosynthesis. Ceramide (Cer) may derive either from dihydrosphingosine (dhSph) through the dihydroceramide desaturase (DES) or from the sphingosine (Sph) by CerS1/2. Ceramidase (CDase) converts Cer in sphingosine (Sph), which, in turn, produces S1P through phosphorylation by sphingosine kinase-1 (SPHK1). SPHK1 may also generate dhS1P from dhSph. Quantitative PCR (qPCR) analysis of CDase **(C)**, CerS1/2 **(D,E)**, SPHK1 **(F)**. *N* = 5 for each group of mice. Data are represented as mean ± SD. *p < 0.05; **p < 0.01 (Unpaired *t*-test).

Here, we investigated whether the overall therapeutic effectiveness of K6PC-5 might be associated with an overall modulation of S1P metabolism. Semi-quantitative analysis by dot blot, performed with a commercial antibody, that specifically detects S1P (Supplementary Figure S3, O’Brien et al., 2009), suggests an increase in the levels of the lipid in the striatal tissues of R6/2 mice after K6PC-5 treatment (Figure 2A). Additionally, with the aim of supporting the findings obtained by dot blot, the expression of enzymes (CerS1, CerS2 and CDase), normally involved in the metabolism of ceramide (Figure 2B), which serves as a precursor of S1P, was investigated.

Chronic administration of K6PC-5 increased the expression of all three enzymes (Figures 2C–E) in striatal tissues from HD mice. Interestingly, mRNA levels of SPHK1 were also increased after treatment (Figure 2F).

**Figure 3:**
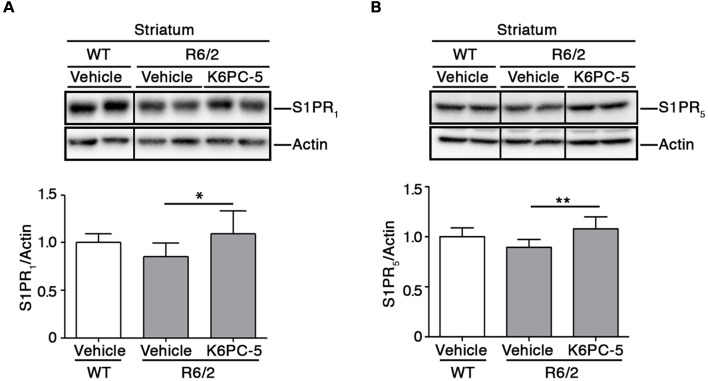
K6PC-5 increases the expression of S1P receptors in the striatum of R6/2 mice. Representative cropped western blotting and densitometric analysis of S1PR_1_ **(A)** and S1PR_5_ **(B)** levels in striatal tissues from vehicle-treated WT and vehicle-and K6PC-5-treated R6/2 mice at 11 weeks of age. Non-adjacent samples were separated by a black line. S1PR_1_: vehicle-treated WT mice, *N* = 6; vehicle-treated R6/2 mice, *N* = 7; K6PC-5-treated R6/2 mice *N* = 5. S1PR_5_: *N* = 7 for each group of mice. Data are represented as mean ± SD. *p < 0.05; **p < 0.01 (One Way ANOVA with Tukey post-test).

Furthermore, K6PC-5 treatment correlated with higher protein levels of S1PR_1_ and S1PR_5_, two receptors of S1P, mostly abundant in the brain (Soliven et al., 2011; Figures 3A,B).

### K6PC-5 Evokes the Activation of Pro-survival Pathways in the Striatum of HD Mice

**Figure 4:**
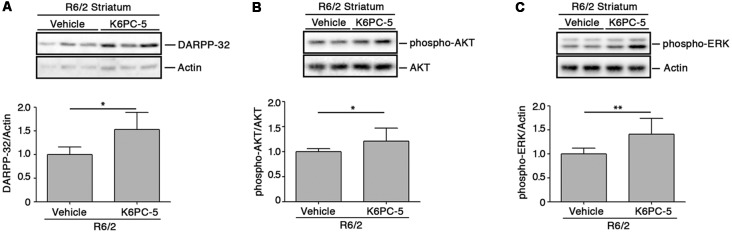
K6PC-5 evokes the activation of pro-survival pathways in the striatum of R6/2 mice. Representative cropped immunoblotting and densitometric analysis of dopamine- and cAMP-regulated protein 32 (DARPP-32; **A**) phospho-AKT/AKT **(B)** and phospho-ERK/Actin **(C)** levels in striatal tissues from vehicle- and K6PC-5-treated R6/2 mice. Phospho-AKT and phospho-ERK: vehicle-treated R6/2 mice, *N* = 8; K6PC-5-treated R6/2 mice, *N* = 5. DARPP-32: vehicle-treated R6/2 mice, *N* = 6; K6PC-5-treated R6/2 mice, *N* = 5. Data are represented as mean ± SD. *p < 0.05; **p < 0.01 (Unpaired *t*-test).

K6PC-5 has previously been demonstrated to evoke the activation of pro-survival kinases AKT and ERK in multiple HD *in vitro* models, including human iPSC-derived HD neurons (Di Pardo et al., 2017a). Here, we explored the potential that K6PC-5 may have in serving as a neuroprotective agent *in vivo*. Administration of K6PC-5 in R6/2 mice was associated with increased levels of striatal DARPP-32 (Figure 4A), a specific marker of medium spiny neurons (Matamales et al., 2009), whose downregulation classically correlates with neurodegeneration in HD (Ehrlich, 2012). Also, K6PC-5 stimulates the phosphorylation of either AKT or ERK, two “classical effectors” whose activation is a prerequisite for neuronal survival also *in vivo* (Figures 4B,C).

### K6PC-5 Increases Levels of Brain Derived Neurotrophic Factor (BDNF) in Both Striatum and Cortex of R6/2 Mice

Defective expression of BDNF has widely been associated with several brain disorders including HD (Zuccato and Cattaneo, 2009). Developing small molecules to target and/or modulate BDNF production has largely been proposed over the past years (Zuccato and Cattaneo, 2009).

**Figure 5:**
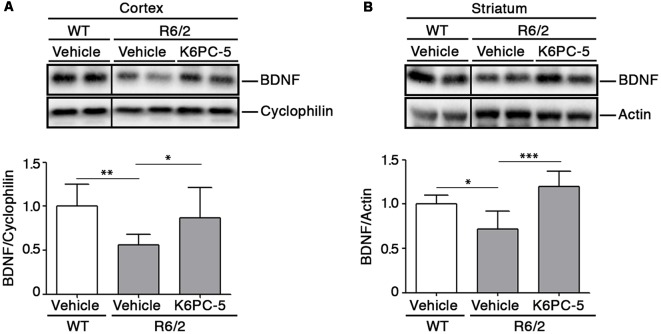
K6PC-5 promotes the elevation of brain derived neurotrophic factor (BDNF) levels in brain tissues from R6/2 mice. Representative cropped western blotting’s and densitometric analysis of BDNF protein in cortical **(A)** and striatal **(B)** tissues from the vehicle-treated WT and vehicle- and K6PC-5-treated R6/2 mice at 11 weeks of age. Cortex: *N* = 9 for each group of mice. Striatum: vehicle-treated WT mice, *N* = 5; vehicle-treated R6/2 mice, *N* = 5; K6PC-5-treated R6/2 mice, *N* = 6 Data are represented as mean ± SD. *p < 0.05; **p < 0.01; ***p < 0.0001 (One Way ANOVA with Tukey post test).

Previous evidence from our group indicates that modulation of S1P axis stimulates the elevation of BDNF levels that are commonly associated with pro-survival effect both in *in vitro* and *in vivo* HD models (Di Pardo et al., 2014, 2018). Interestingly, after administration of K6PC-5, levels of BDNF protein were found markedly elevated in both cortical tissues and in the striatum of R6/2 mice (Figures 5A,B).

### K6PC-5 Reduces mHtt Aggregation in Brain Tissues From R6/2 Mice

**Figure 6:**
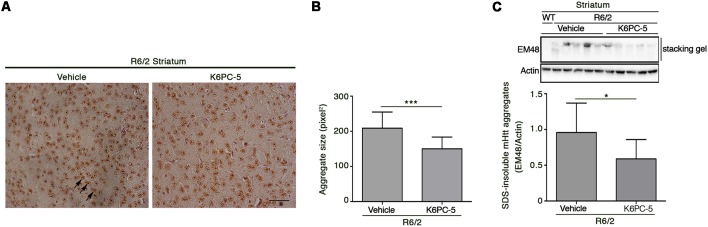
Administration of K6PC-5 ameliorates neuropathology in R6/2 mice. Representative micrograph **(A)** and semi-quantitative analysis of EM48-immunoreactive mutant huntingtin (mHtt) aggregate area **(B)** in the striatum of vehicle- and K6PC-5-treated R6/2 mice at 11 weeks of age. Arrows indicate mHtt aggregates. Scale bar in represents 100 μm. HD, *N* = 4 + 4. Values are represented as mean ± SD. ***p < 0.001 (Unpaired *t*-test). **(C)** Representative cropped immunoblotting of EM48-positive SDS-insoluble mHtt aggregates in striatal lysate from vehicle- and A-K6PC-5-treated R6/2 mice at 11 weeks of age. Vehicle-treated R6/2 mice, *N* = 10; K6PC-5-treated R6/2 mice, *N* = 8. Values are represented as mean ± SD. *p < 0.05 (Unpaired *t*-test).

Formation of mHtt aggregates is a pathological hallmark that may conceivably cause neuronal dysfunction in HD (Sánchez et al., 2003; Arrasate et al., 2004). Here we tested the possibility that K6PC-5 treatment may lower mutant Htt toxicity likely modulating its aggregation. Immunohistochemical staining for EM48 highlighted that the size of mHtt aggregates was significantly reduced in both the striatum and cortex of K6PC-5-treated R6/2 mice compared with the controls (Figures 6A,B and Supplementary Figure S4). This finding was also confirmed by immunoblotting analysis which showed a decrease of EM48-positive SDS-insoluble aggregates in striatal protein lysates (Figure 6C).

### Reduction of mHtt Aggregation Is Associated With Modulation of Autophagic Markers

**Figure 7:**
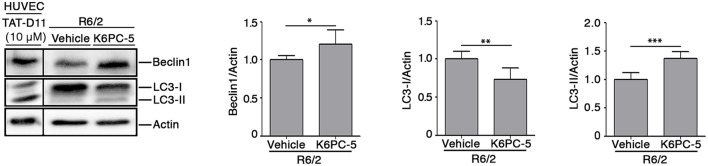
Administration of K6PC-5 modulates autophagic flux in the striatum of R6/2 mice. Representative cropped immunoblotting and densitometric analysis of Beclin1 and LC3-I/-II autophagic markers in striatal tissues from vehicle- and K6PC-5-treated R6/2 mice at 11 weeks of age. Cell lysate from HUVEC, treated with the autophagy inductor TAT-D11 (10 μM; Shoji-Kawata et al., 2013) was used as positive control. All samples were run on the same gel. Non-adjacent samples were separated by a black line. Vehicle-treated R6/2 mice, *N* = 6; K6PC-5-treated R6/2 mice, *N* = 6. Values are represented as mean ± SD. *p < 0.05; **p < 0.01; ***p < 0.001 (Unpaired *t*-test).

Evidence indicates that S1P may regulate autophagy in different experimental conditions and cell types, including neuronal cells (Karunakaran and van Echten-Deckert, 2017). Increased autophagy has been reported as a molecular mechanism associated with clearance of mHtt aggregation in multiple HD pre-clinical models (Yamamoto et al., 2006; Martin et al., 2015). Here, in order to investigate any correlation between the reduction of mHtt aggregates after K6PC-5 administration and autophagy, protein expression of Beclin1 and LC3, two of the most widely used cellular autophagic markers (Moulis and Vindis, 2017), in striatal tissues from HD mice, was assessed. K6PC-5 administration was associated with an increased expression of Beclin1 (Figure 7), whose elevation has been reported to be correlated with an increased autophagic flux (Moulis and Vindis, 2017), with decreased levels of LC3-I and elevation of LC3-II (Figure 7), further reinforcing the hypothesis of high autophagic activity after treatment.

## Discussion

The complexity and variety of aberrant molecular pathways that are commonly associated with HD have long hampered the development of effective therapies for this disease. Over the last few years, the discovery that sphingolipid metabolism can play a key role in HD pathogenesis and can affect many of the cellular pathways associated with mHtt toxicity, has opened the door to novel potential therapeutic strategies that specifically target the central lipid homeostasis which is tightly regulated to maintain neuronal structure and function (Di Pardo and Maglione, 2018a, b).

Alterations of lipid metabolism have been frequently associated with HD, however the recognition of perturbed sphingolipid metabolism has only very recently become evident in the disease (Maglione et al., 2010; Pirhaji et al., 2016, 2017; Di Pardo et al., 2017a,b).

Aberrant sphingolipid metabolism, with reduced bioavailability of the bioactive lipid S1P, has been reported in multiple HD settings (Pirhaji et al., 2016, 2017; Di Pardo et al., 2017a,b) and, interventions aimed at modulating S1P metabolism/axis have repeatedly been proposed as alternative therapeutic options in the disease (Di Pardo et al., 2014, 2018; Pirhaji et al., 2016; Di Pardo and Maglione, 2017, 2018a; Moruno-Manchon et al., 2017, 2018).

In this study, we reported for the first time, that stimulation of SPHK1 activity is beneficial in a HD animal model. Administration of K6PC-5, a selective activator of SPHK1, exerted an overall beneficial effect on the disease phenotype in R6/2 mice. Besides preventing motor dysfunction, K6PC-5 treatment was associated with conserved body weight and expanded lifespan in this mouse model.

Our results are coherent with a real modulation of S1P pathways in HD, however, the precise molecular mechanisms behind such a therapeutic action remain to be further elucidated. Metabolism of S1P is very complex with several points of regulation including the implication of receptors. Thus, it is conceivable that any modulation of sphingolipid content may determine a global rearrangement of the metabolic pathways.

Reduced content of S1P has previously been associated with HD pathogenesis (Di Pardo et al., 2017a; Pirhaji et al., 2017) and its elevation after K6PC-5 administration likely lies behind the therapeutic action of the compound in R6/2 mice.

**Figure 8:**
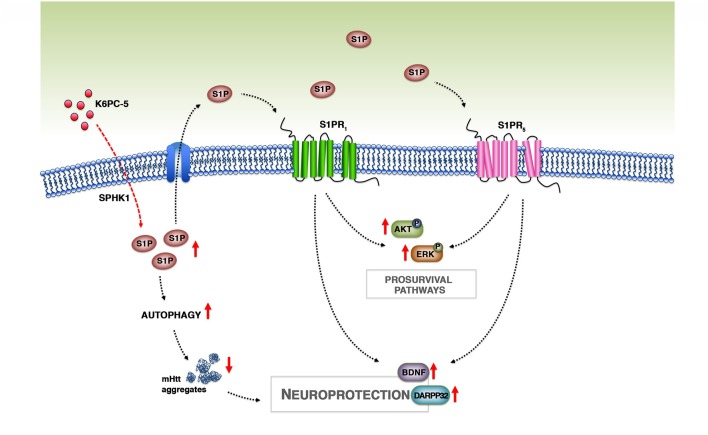
Working model. Treatment with K6PC-5 stimulates the activity of SPHK1 with a subsequent increase in the levels of S1P. S1P may act either intracellularly, by stimulating autophagy and reducing mHtt aggregation, or extracellularly by stabilizing S1PR expression and activating pro-survival pathways.

The beneficial effect of K6PC-5 seems to be mediated by a number of different molecular mechanisms, however, although only speculative, we believe that an incremented bioavailability of S1P after the treatment may play a pivotal role (Figure 8). Changes in the expression of ceramide-metabolizing enzymes (CerS1/2 and CDase) and the incremented expression of SPHK1, probably due to a positive feedback mechanism, may presumably indicate a shift in the sphingolipid rheostat away from ceramide and in favor of S1P. Considering the limitations of the technique applied for determining S1P levels in this study, we believe that qPCR findings represent an indirect indication of the ability of the compound to modulate sphingolipid metabolism and conceivable increase S1P levels in brain tissues.

K6PC-5 increased protein levels of S1PR_1_ and S1PR_5_, the latter of which has recently emerged as a therapeutic target in R6/2 mice (Di Pardo et al., 2018). Elevation in the expression of both receptors may be conceivable attributable to higher S1P bioavailability that may somehow stabilize the proteins, promoting then the phosphorylation of pro-survival kinases AKT and ERK. This is particularly important since the alteration in either AKT or ERK seems to be crucial in the pathogenesis of HD and their activation has been proposed as a reasonable event to suppress disease progression (Rai et al., 2019).

Evidence also indicates that increased bioavailability of BDNF predicts benefits in R6/2 mice (Giralt et al., 2011; Giampà et al., 2013). Administration of K6PC-5 in these mice increased levels of BDNF in the cortex and importantly also in the striatum, which normally rely on cortex-synthetized neurotrophin. Thus, it is plausible that K6PC-5 favors the anterograde transport of the BDNF from the cortex to the striatum, which in turn is protected from the loss of striatal neurons, as revealed by increased levels of DARPP-32.

Additional molecular mechanisms underlying the beneficial effect of K6PC-5 may depend on the correlation existing between the S1P biosynthetic enzyme, SPHK1, and the autophagic flux (Lavieu et al., 2006; Moruno Manchon et al., 2015). Coherently, reduced levels and/or activity of SPHK1 have been described to impair endocytic membrane trafficking pathways (Shen et al., 2014) and to inhibit neuronal autophagy in different neurodegenerative conditions (Lee et al., 2014). Its over-expression, instead, stimulates autophagic flux in cells including primary neurons (Lavieu et al., 2006; Moruno Manchon et al., 2015).

In this study, for the first time, we demonstrated that stimulation of SPHK1, by K6PC-5, triggers autophagy *in vivo* in R6/2 mice. This is absolutely in line with the evidence that this enzyme is necessary for promoting the formation of pre-autophagosoms in primary neurons (Moruno Manchon et al., 2015). Change in the expression of Beclin1 and the conversion of LC3-I to LC3-II, all markers classically associated with autophagosomal organization, are clear signs of active autophagic machinery *in vivo*.

Evidence indicates that autophagy may represent a potential therapeutic target in HD pre-clinical models (Martin et al., 2015) and eventually influence the disease course in HD patients (Metzger et al., 2013). Although in this study, we cannot establish any direct correlation between increased autophagy and reduction of mHtt aggregates, we can speculate that clearance of protein aggregates after administration of K6PC-5 may be associated with boosted autophagy flux. Further studies are necessary to fully clarify this molecular mechanism, however recent evidence demonstrates that overexpression of SPHK1 promotes clearance of mutant Htt exon-1 construct *in vitro* (Moruno Manchon et al., 2015) and suggests a possible link between increased autophagy and reduced mHtt aggregation.

Although we did not have any direct evidence of the ability of K6PC-5 to penetrate the brain, our findings clearly indicate that the compound was able to modulate brain homeostasis. From our perspective, stimulation of SPHK1 exerts a larger array of biological effects by acting on both intracellular (stimulation of the defective sphingolipid metabolism and induction of autophagy) and extracellular pathways (receptor axis and related signaling), with respect to the stimulation of S1P receptors.

What makes our findings attractive is the evidence that S1P metabolism may represent a target for the discovery of novel therapeutic strategies in HD, especially given that drugs working through its related pathways are already in clinical trials for different other pathological conditions (Kunkel et al., 2013; Gonzalez-Cabrera et al., 2014; O’Sullivan and Dev, 2017; Di Pardo and Maglione, 2018a).

In conclusion, with this study we provide further evidence that modulation of S1P metabolism may represent a strong paradigm for pursuing the development of novel and more targeted therapeutic strategy by which it is possible to restore normal sphingolipid metabolism and stimulate S1P axis, which in turn may activate pro-survival pathways and reduce mHtt aggregation with a consequent preservation of neuronal homeostasis in HD brains (Figure 8).

## Ethics Statement

All experimental procedures were approved by the IRCCS Neuromed Animal Care Review Board and by “Istituto Superiore di Sanità” (ISS permit number: 1163/2015-PR) and were conducted according to EU Directive 2010/63/EU for animal experiments.

## Author Contributions

VM and ADP conceived and designed the study, jointly directed the study and co-wrote the manuscript. ADP supervised all *in vivo* experiments. SC, LC, MM and SG managed animal colonies and performed all *in vivo* analyses. FM, EA, GP performed all the biochemical and histological experiments and made manuscript revisions. SJ, B-MP and BP provided K6PC-5 technology and the PAMPA assay results. All the authors analyzed and discussed the data, revised and approved the manuscript.

## Conflict of Interest Statement

B-MP was employed by company NeoPharm USA Inc. BP was employed by company Dr. Raymond Laboratories Inc., USA. The remaining authors declare that the research was conducted in the absence of any commercial or financial relationships that could be construed as a potential conflict of interest.
