# Supplementary material for: Stimulation of Sphingosine Kinase 1 (SPHK1) Is Beneficial in a Huntington’s Disease Pre-clinical Model
Source: Front Mol Neurosci. 2019 Apr 24;12:100. doi: 10.3389/fnmol.2019.00100 (PMC6491579; doi:10.3389/fnmol.2019.00100)
Supplement: Supplementary file 1 [file Data_Sheet_1.PDF]

Supplementary Information

Stimulation of Sphingosine Kinase 1 (SPHK1) is beneficial in a Huntington's disease pre-clinical model

Di Pardo A[1*], Pepe G[1], Castaldo S[1], Marracino F[1], Capocci L[1], Amico E[1], Madonna M[1], Giova S[1], Jeong SK[2], Park BM[3], Park BD[4] and Maglione V[1*].

***Correspondence to:**

Vittorio Maglione

E-mail: vittorio.maglione@neuromed.it

and

Alba Di Pardo

E-mail: alba.dipardo@neuromed.it

[1]IRCCS Neuromed, Pozzilli, Italy

[2]Department of Cosmetic Science, Seowon University, Cheongju, Korea

[3] NeoPharm USA Inc. Engelwood Cliffs, New Jersey, USA

[4] Dr. Raymond Laboratories, Inc, Englewood cliffs, New Jersey, USA

Supplementary Figure 1. Pilot Study (0.1 and 0.05 mg/kg K6PC-5). Treatment with 0.05 mg/kg K6PC-5 ameliorates motor performance in R6/2 HD mice as assessed by Rotarod test. N=3 for each group of mice.

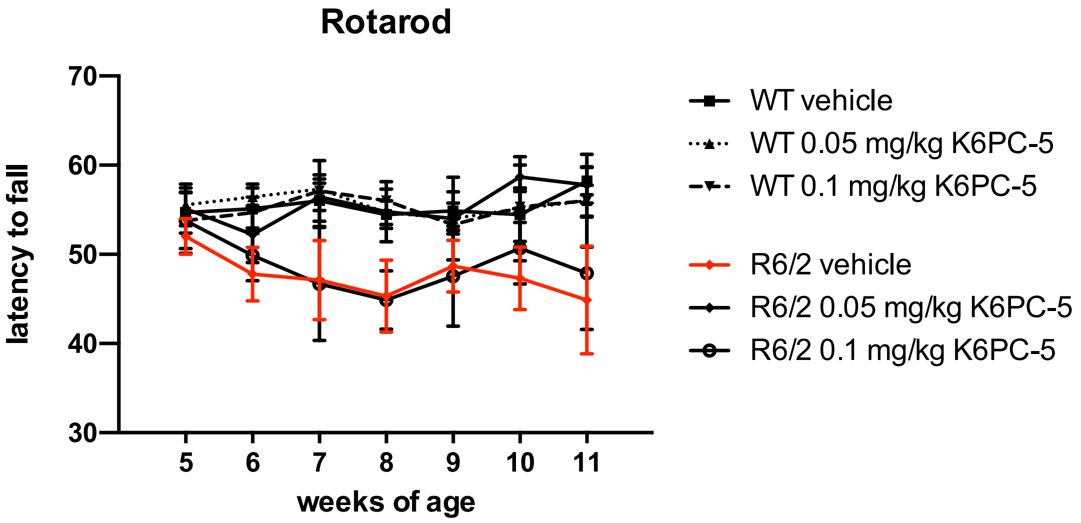

Supplementary Figure 2. PAMPA Assay. K6PC-5 shows high BBB permeability as assessed *in-vitro*.

Permeability classification:

- **high** for $P_e > 0.4 \times 10^{-6}$ cm/sec
- **low** for $P_e < 0.4 \times 10^{-6}$ cm/sec

CNS classification:

- **CNS+** for $P_e > 10^{-6}$ cm/sec
- **CNS-** for $P_e < 10^{-6}$ cm/sec.

Solubility test for PAMPA buffer system

Test concentration	50μM	25μM	12.5μM
K6PC-5	+	-	-

(+: slight precipitation observed, -: no precipitation observed)

PAMPA analysis data summary

Test compound	Concentration	P_e (10^{-6}cm/sec)	BCS code
Progesterone	50μM	46.699	High (CNS+)
Lidocaine	50μM	24.698	High (CNS+)
Theophylline	50μM	0.174	Low (CNS-)
**K6PC-5**	**25μM**	**12.733**	**High (CNS+)**

Supplementary Figure 3. Anti-S1P antibody specifically recognizes S1P. Different amount of purified lipid species S1P (SIGMA Cat. N. 73914-5mg) and Sphingomyelin (SIGMA, Cat. N. 50756-50mg) were spotted on nitrocellulose membrane. Membrane was then incubated with the anti-S1P antibody (LT1002) (1:500) (Echelon Biosciences, Cat. N. Z-P300). A monoclonal anti-mouse HRP-conjugated antibody (Santa Cruz, Cat. N. sc-2005) was used as secondary antibody. S1P-immunopositive spots were visualized by ECL Plus (GE Healthcare).

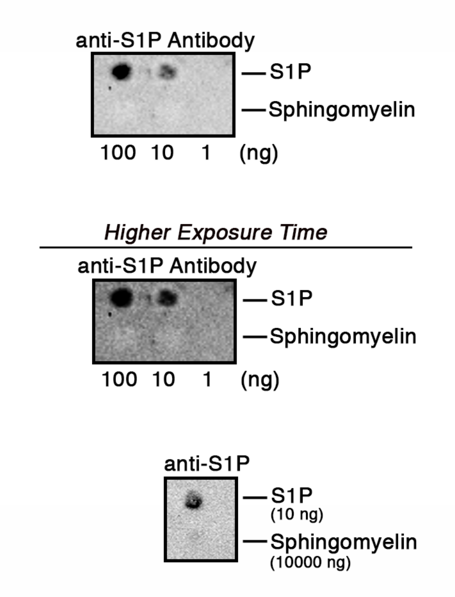

Supplementary Figure 4. Treatment with K6PC-5 reduced mHtt aggregation in the cortex of R6/2 mice. Arrows indicate mHtt aggregates. Scale bar in represents 50μm. N=4 for each group of mice. *, $p<0.05$ (Unpaired *t*-test).

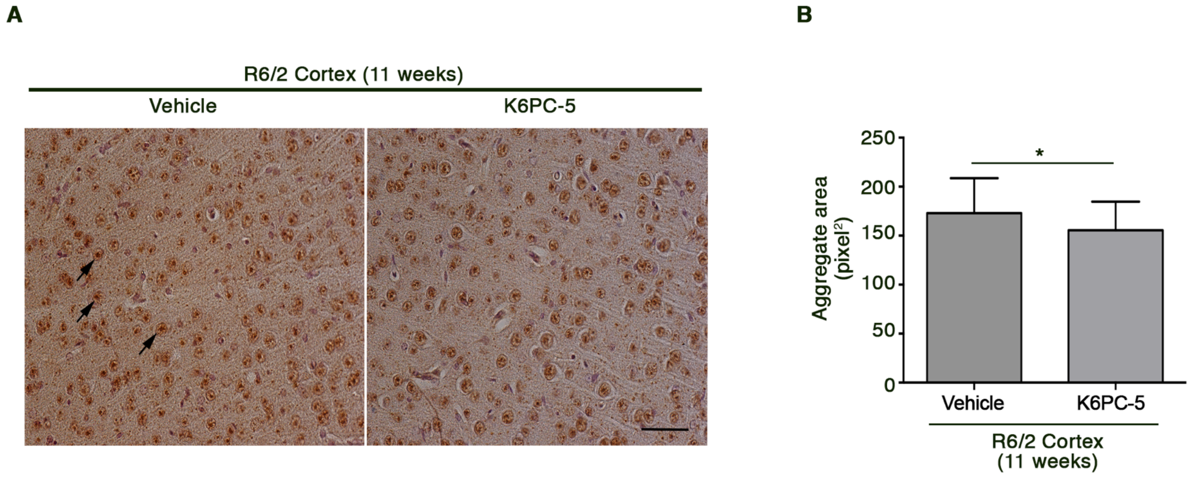

Supplementary Methods

Experimental procedure for S1P Dot Blotting. After protein quantitation (see Methods in the main text), each single tissue lysate (i.e. sample 1) was spotted on nitrocellulose membrane by using a multichannel pipet (8 spots for each sample).

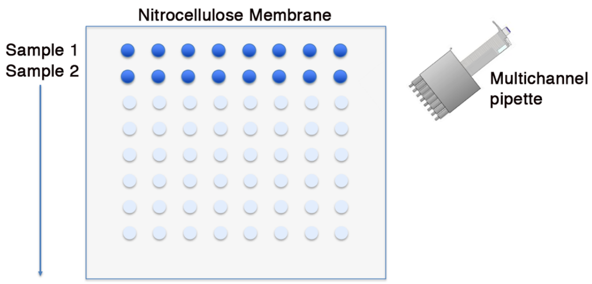

The membrane was cut into two equal parts and each of them was incubated with the specific antibody as shown below.

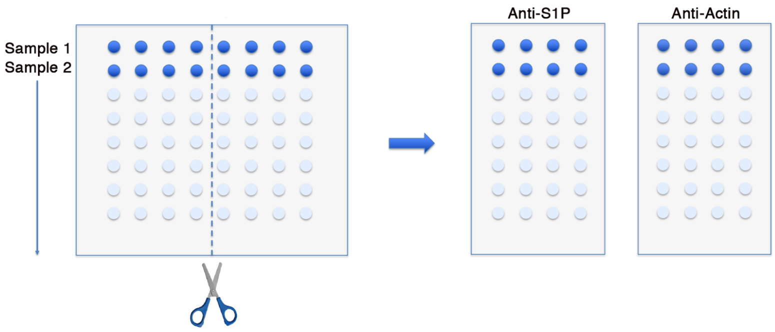

S1P- and Actin-immunopositive spots were visualized by ECL Plus (GE Healthcare) and quantitated with Image Lab Software (Bio-Rad Laboratories).

Graphical representation of the results was obtained by PRISM 6 software. For each single sample, the value of S1P/Actin was calculated considering the average of all four spots.

PAMPA Assay

Experimental condition

PAMPA	Double sink (Pion, BBB PAMPA)
Assay format	96-well plate
Reference compound	Theophylline, Lidocaine, Progesterone
Number of replicates	3
Stock soln of compound	10mM in DMSO
Test pH	7.4
Test concentration	Reference (50μM), Test (25μM)
Incubation time	4hr
Analysis method	UV detection

Experimental methods

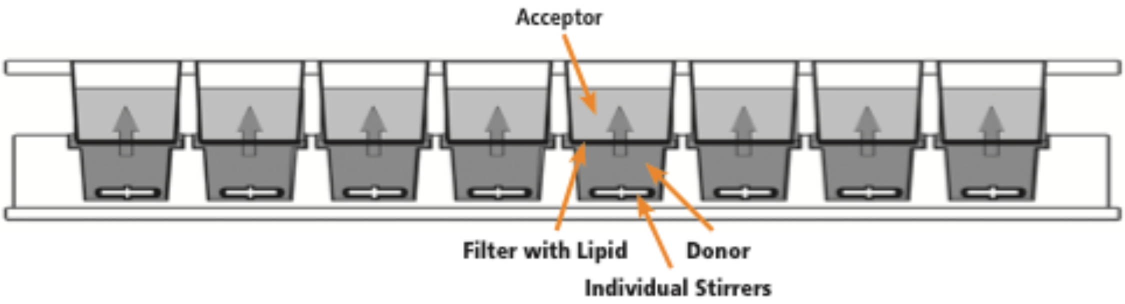

UV blank plate preparation:

150 μl of buffer (pH 7.4) was added to UV blank plate and the blank spectrum was read using spectrophotometer in scanning mode from 230 nm to 498 nm using PION PAMPA EXPLORER (Version 3.8).

Reference UV plate preparation

150 µl of diluted 50 µM stock solutions was added to the UV reference plate and the reference spectrum will be read using spectrophotometer in scanning mode from 190 nm to 500 nm using PION PAMPA EXPLORER (Version 3.8).

Sample plate preparation and incubation

180 µl of the diluted 50 µM stock was added to the donor (bottom) plate of the PAMPA sandwich plate.

5 µl of the BBB-1 lipid solution was painted at the bottom side of the PAMPA acceptor plate by turning the plate upside down. The painted acceptor plate was placed on top of the donor plate in its normal position.

200 µl of the Acceptor sink buffer was added to the top acceptor plate.

The PAMPA sandwich plate will be kept for 4 hours incubation at 25°C.

After 4 hours incubation, 150 µl was aliquoted from acceptor plate and added in the UV plate and read by using the PAMPA pION software as Acceptor spectrum.

After reading the acceptor plate 150 µl of the donor solution was added to UV plate and read by PION PAMPA EXPLORER (Version 3.8).

References

[1] Kansy, M.; Senner, F.; Gubernator, K. J. Med. Chem. 1998, 41, 1007-1010.

[2] Avdeef, A. High-throughput measurements of solubility profiles. In: Testa, B., van deWaterbeemd, H., Folkers, G., Guy, R. (Eds.), Pharmacokinetic Optimization in Drug Research, Verlag Helvetica Chimica Acta: Zurich and Wiley - VCH: Weinheim, 2001, pp.305-326. (Part 1 in PAMPA series)

[3] Avdeef, A.; Strafford, M.; Block, E.; Balogh, M.P.; Chambliss, W.; Khan, I. Drug Absorption In Vitro Model: Filter-Immobilized Artificial Membranes. 2. Studies of the Permeability Properties of Lactones in Piper methysticum Forst. Eur. J. Pharm. Sci. 2001, 14, 271-280. (Part 2 in PAMPA series)
